# Impact of 3D Laparoscopy on Surgical Efficiency and Ergonomics in Elective Cholecystectomy: A Prospective Randomized Comparative Study

**DOI:** 10.7759/cureus.106108

**Published:** 2026-03-30

**Authors:** Navneeth Sankar S, Ashwani Kumar, Jaswinder Singh, Sanjeev Gupta, Dinesh Pasi, Parth Dhamija

**Affiliations:** 1 Department of General Surgery, Government Medical College Patiala, Patiala, IND

**Keywords:** 2d laparoscopy, 3d laparoscopy, cholecystectomy, laparoscopic cholecystectomy, surgical ergonomics

## Abstract

Introduction

Laparoscopic cholecystectomy is the standard treatment for symptomatic cholelithiasis. Although 3D laparoscopy may improve depth perception, its effects on efficiency, ergonomics, and complication rates in comparison to 2D systems remain unclear.

Methods

In this prospective, single-blinded, randomized comparative study, 100 patients underwent elective laparoscopic cholecystectomy at the Department of General and Minimal Access Surgery in a tertiary care teaching hospital in North India. Patients were randomized equally to 3D (Group A) or 2D laparoscopy (Group B). Exclusion criteria included prior major abdominal surgery, coagulopathy, or pregnancy. Total performance time, surgeon’s subjective depth perception (scale 1-5), strain scores (eye, wrist/hand, neck/back, dizziness/headache), intraoperative complications, conversion rates, and hospital stay were documented through surgical records and postoperative questionnaires.

Results

The total performance time was significantly shorter in the 3D group (46 min) compared to the 2D group (50 min; p=0.0074). The median depth perception score was higher in the 3D group (5.0 vs. 2.0; p<0.001). Wrist/hand strain was lower in the 3D group (median 2.0 vs. 3.0; p=0.0117), while eye strain (4.0 vs. 3.0; p=0.0021) and dizziness/headache (4.0 vs. 2.0; p<0.001) were greater. There were no significant differences in neck/back strain (p=0.81), rates of gallbladder rupture (10% vs. 12%; p=0.76), liver bed bleeding (10% vs. 14%; p=0.76), biliary injury (none), conversion rates (2% in both), or hospital stay (2.06 days each; p=0.78).

Conclusions

3D laparoscopy enhances operative efficiency and depth perception while reducing wrist/hand strain. However, increased eye strain and dizziness warrant further technological optimization to improve the overall ergonomic profile for surgeons.

## Introduction

Cholelithiasis, widely known as gallstone disease, is characterized by the development of calculi within the gallbladder [1]. While a significant portion of the affected population remains asymptomatic, patients who develop symptoms often present with abdominal pain, nausea, and vomiting, or may progress to severe sequelae such as acute cholecystitis, pancreatitis, and gallbladder perforation [2]. Management strategies depend on the severity of the presentation; however, for symptomatic cholelithiasis, cholecystectomy is widely accepted as the definitive and most common treatment [3]. While open surgery was historically the norm, laparoscopic cholecystectomy (LC) has evolved to become the modern gold standard for treatment [4].

Standard LC is typically performed using a four-port technique under two-dimensional (2D) visualization [5]. Although 2D laparoscopy has revolutionized the field of minimally invasive surgery, it possesses inherent technical limitations [6]. The primary disadvantage is the reliance on a flat monitor, which results in a loss of depth perception, making it challenging for surgeons to accurately gauge distances and spatial relationships within the surgical field [7,8]. Furthermore, this lack of stereoscopic cues can lead to visual misperception, where the 2D image fails to provide sufficient context for accurate intraoperative decision-making [3].

The introduction of 3D laparoscopy represented a major technological milestone, designed to overcome the constraints of traditional 2D systems by restoring depth perception and spatial orientation [7,9]. Evidence suggests that 3D systems significantly improve surgical accuracy and reduce operative duration by providing superior spatial awareness compared to 2D systems [10]. This technology restores binocular depth perception, which is instrumental in enhancing surgical precision [11]. By delivering improved visual feedback, these systems may facilitate faster procedural completion without compromising patient safety standards [12].

Despite these potential benefits, the widespread implementation of 3D laparoscopy has been gradual, often attributed to the learning curve associated with the technology and the significant costs of implementation [10]. Additionally, the requirement for prolonged use of specific 3D glasses and potential issues with monitor positioning can cause visual disturbances; this economic burden requires justification for routine clinical application [13]. Consequently, a debate persists regarding the true efficiency and superiority of 3D over 2D laparoscopy; while many experts consider it a vital improvement for precision, others argue it offers no significant advantage and may even increase surgeon fatigue [14]. This study aims to provide a randomized comparison of these modalities, specifically focusing on operative efficiency and surgeon ergonomics.

## Materials and methods

Study design and setting

This prospective, single-blinded, randomized comparative study was conducted at the Department of General and Minimal Access Surgery in a tertiary care teaching hospital in North India. The study was carried out from January 2024 to December 2024 and concluded upon the completion of the required sample size. The protocol was approved by the Institutional Ethics Committee, and the study adhered to the ethical principles outlined in the Declaration of Helsinki.

Participants and eligibility

The study population consisted of patients diagnosed with symptomatic cholelithiasis scheduled for elective laparoscopic cholecystectomy. A sample size of 100 patients (50 per group) was calculated based on previous studies to detect a statistically significant difference in total performance time, assuming a power of 80% and a two-sided significance level of 0.05 (alpha). Inclusion criteria comprised patients aged between 18 and 70 years who were fit for general anesthesia and provided written informed consent. Patients were excluded if they had a history of previous major abdominal surgery, coagulopathy, or were pregnant. Additionally, those scheduled for planned additional abdominal procedures, patients outside the specified age range, or those who refused to participate were not included in the study.

Randomization and group allocation

Recruitment occurred twice weekly, with the first eligible consecutive case of the day being enrolled into the study. Following enrollment, participants were assigned to one of two equal groups (1:1 ratio) on the day of surgery using a pre-generated computer random allocation sequence managed by the principal investigator. Group A (n = 50) underwent laparoscopic cholecystectomy using a 3D high-definition (HD) laparoscopic system, while Group B (n = 50) was assigned to a standard 2D-HD setup. While patients were blinded to the group allocation, the operating surgeon could not be blinded due to the requirement of wearing specific 3D polarized glasses for Group A procedures.

Surgical technique

All procedures were performed under general anesthesia utilizing a standardized four-port laparoscopic cholecystectomy technique. A 10-mm umbilical camera port was established first, followed by the insertion of three additional instrument ports under direct vision. The surgical team performed dissection of Calot’s triangle to isolate, clip and divide the cystic duct and cystic artery. The gallbladder was subsequently dissected from the liver bed and removed via the epigastric port. A 16F sub-hepatic drain was inserted in all patients to monitor for postoperative bleeding or bile leak.

Outcome measures and data collection

Outcomes were assessed using objective intraoperative metrics and subjective surgeon-reported questionnaires recorded immediately after each procedure. The primary objective outcome was total performance time, defined as the duration in minutes from Veress needle insertion to gallbladder retrieval. Subjective outcomes included depth perception and surgeon strain. Depth Perception was rated by the operating surgeon on a generic 5-point subjective scale (1 = Very Poor, 5 = Excellent). Surgeon Strain was evaluated across four domains of eye strain, wrist/hand strain, neck/back strain, and dizziness/headache using a non-proprietary 5-point Likert scale (1 = No Strain, 5 = Severe Strain). Intraoperative complications, including gallbladder rupture, liver bed bleeding, biliary injury, and conversion to open surgery, were documented from surgical records.

Statistical analysis

Data compilation was performed using Microsoft Office Excel version 2024 (Microsoft Corp., Redmond, WA) and statistical analysis was conducted using SPSS version 21.0 for Microsoft Windows (IBM Corp., Armonk, NY). Continuous variables were described as means with standard deviations (SD). Categorical data were analyzed using the chi-square test or Fisher’s exact test as appropriate. For continuous variables, independent t-tests or Mann-Whitney U tests were utilized based on data distribution. A probability value (p-value) of less than 0.05 was considered statistically significant.

## Results

Demographic characteristics

The study population encompassed a broad age range, with patients distributed across adult age brackets and demonstrating baseline comparability between the two groups. In total, the study included 100 patients randomized equally into two groups. The most prevalent age group was 26-40 years, comprising 18 (36%) patients in Group A (3D) and 22 (44%) patients in Group B (2D). Older adults aged 56-70 years accounted for 11 (22%) patients in Group A and 8 (16%) in Group B. There was no statistically significant difference in the age distribution between the two groups (χ^2^=5.117, p=0.163), ensuring baseline comparability.

Operative efficiency

**Figure 1:**
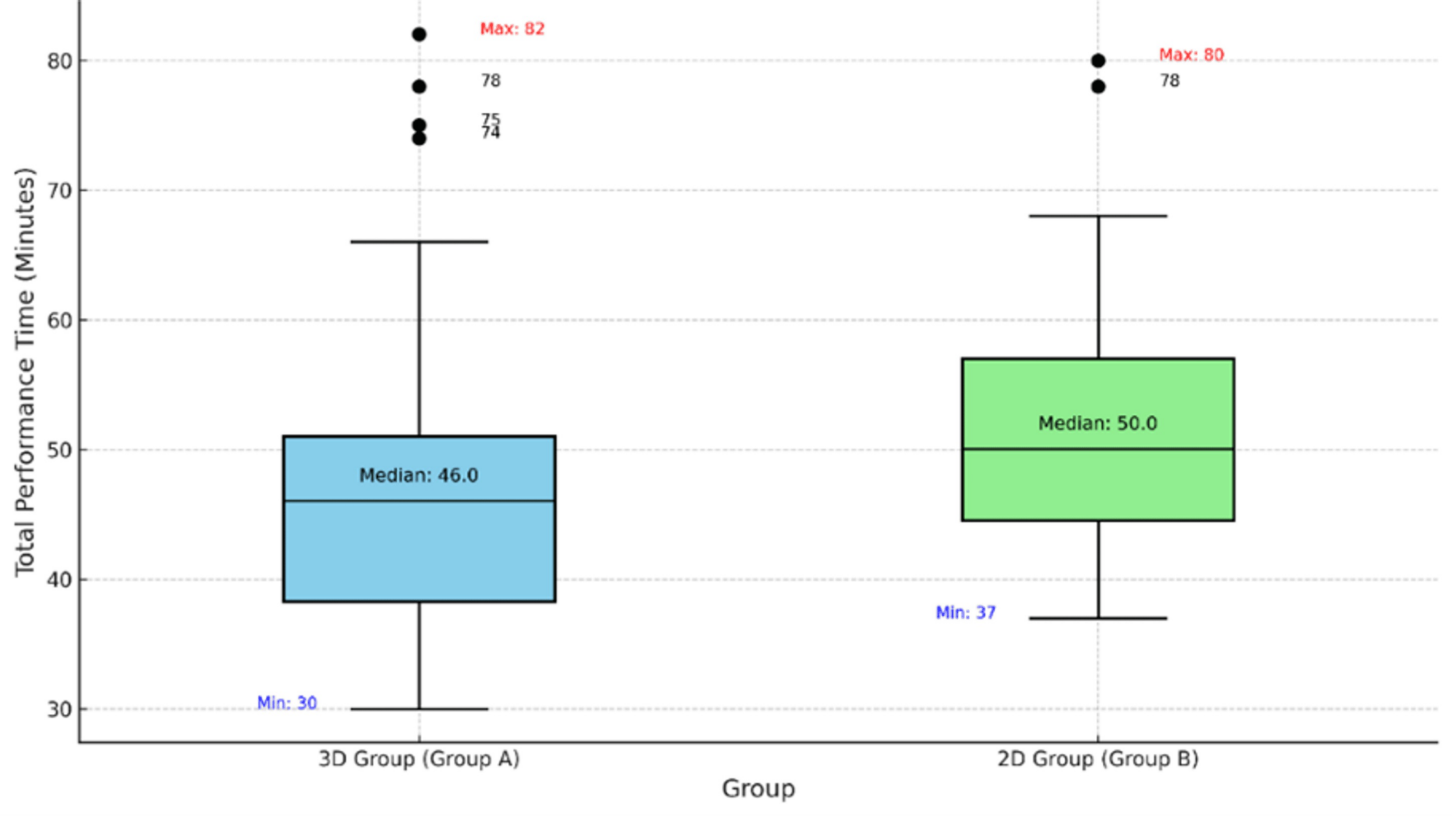
Total Performance Time (in minutes)

The median total performance time was significantly shorter in the 3D group compared to the 2D group. Group A (3D) recorded a median time of 46.0 minutes (IQR: 38-51), whereas Group B (2D) recorded a median of 50.0 minutes (IQR: 45-57) (Figure 1).

This reduction in total performance time was statistically significant (Mann-Whitney U = 862, p = 0.0074). Outliers were observed in both groups, with maximum durations reaching 82 minutes in the 3D group and 80 minutes in the 2D group. Clinical review of these cases revealed that the extended durations were primarily due to increased case complexity rather than the visualization system used. In the 3D group, the primary outliers involved gallbladder rupture (n=3), liver bed bleeding (n=4), and one conversion to open surgery. Similarly, both outlier cases in the 2D group involved gallbladder rupture and intraoperative bleeding. These findings suggest that while 3D laparoscopy reduces average total performance time, acute complications remain the primary driver of significantly prolonged surgical duration. 

Depth perception and ergonomics

**Figure 2:**
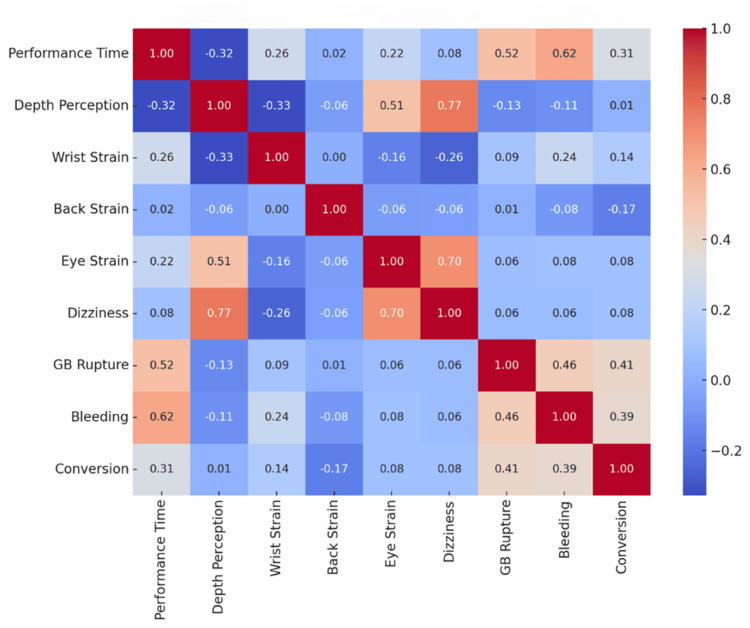
Correlation Heatmap of Surgical Variables

Surgeon-reported depth perception was significantly higher in the 3D group, with a median score of 5.0 (IQR: 4.0-5.0) compared to 2.0 (IQR: 2.0-3.0) in the 2D group (U=210.5, p<0.001). In terms of physical ergonomics, the 3D group reported significantly lower wrist and hand strain (median 2.0 vs. 3.0 (U=885.0, p=0.0117)). However, regarding ocular ergonomics, the 3D group reported significantly higher scores for eye strain (median 4.0 vs. 3.0 (U=812.5, p=0.0021) and dizziness/headache (median 4.0 vs. 2.0 (U=314.0, p<0.001)). There was no significant difference in neck and back strain between the groups (median 3.0 vs. 3.0 (U=1218.0, p=0.813)). The correlation between these surgical variables is presented in Figure 2.

Intraoperative complications

There was no statistically significant difference in the overall complication rates between the two groups. Gallbladder rupture occurred in 5 (10%) patients in the 3D group and 6 (12%) patients in the 2D group (χ^2^=0.102, p=0.76). The majority of ruptures occurred during extraction (40% of ruptures in Group A; 33% in Group B). Liver bed bleeding was observed in 5 (10%) patients in the 3D group and 7 (14%) patients in the 2D group (χ^2^=0.378, p=0.76). No biliary tract injuries were recorded in either group. Conversion to open surgery was required in 1 (2%) patient in each group (p=1.0), typically in cases where both rupture and bleeding were present.

Correlation of complications

An analysis of the correlation between intraoperative complications (Table 1) revealed a moderate positive association between gallbladder rupture and liver bed bleeding (overall ϕ=0.362, χ^2^=13.10, p=0.027).

**Table 1:** Correlation Analysis of Intraoperative Complications (Gallbladder Rupture and Liver Bed Bleeding) Across Study Groups The association between gallbladder rupture and liver bed bleeding was analyzed using the phi (ϕ) coefficient and chi-square (χ^2^) test. While a moderate positive correlation was observed overall (p = 0.027), subgroup analyses did not reach statistical significance due to the low event rate. A p-value of less than 0.05 was considered statistically significant.

Group	Co-occurrence (Bleeding + Rupture)	Bleeding Only	Rupture Only	Neither	Phi (ϕ) Coefficient	Test Statistic (χ^2^)	Significance (p-value)
2D Group (B)	3	4	3	40	0.387	7.49	0.098
3D Group (A)	2	3	3	42	0.345	5.95	0.147
Overall	5	7	6	82	0.362	13.10	0.027

**Figure 3:**
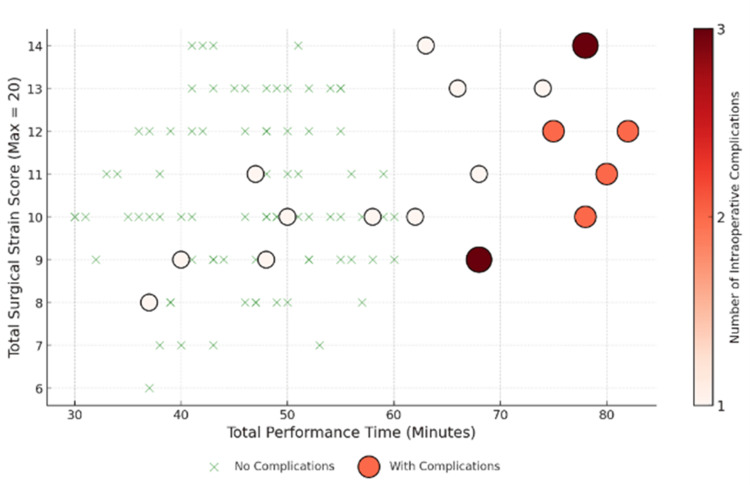
Bubble Chart Illustrating the Relationship Between Total Performance Time, Surgical Strain, and Intraoperative Complications

This suggests that cases involving one complication are significantly more likely to involve the other, likely due to shared technical difficulties during the dissection of the liver bed. Though the subgroup correlations for 2D (ϕ=0.387, χ^2^=7.49, p=0.098) and 3D (ϕ=0.345, χ^2^=5.95, p=0.147) showed moderate associations, they did not reach statistical significance individually due to the low number of events. The relationship between total performance time, surgical strain, and complications is illustrated in the heat map (Figure 3).

Postoperative outcomes

The duration of hospital stay was nearly identical between the two groups, with a mean of 2.06 days and a median of 2.0 days (IQR: 2.0-2.0) for both. No significant difference was found in the length of stay (U=1208.5, p=0.775). Prolonged hospitalizations (>4 days) were exclusively associated with patients who experienced major intraoperative complications or conversion to open surgery.

## Discussion

Operative efficiency

The total performance time was significantly shorter in the 3D group compared to the 2D group (p = 0.0074). This finding aligns with studies by Bilgen et al. (2013) [10] and Currò et al. (2015) [15], which reported reduced total performance time due to improved spatial awareness and depth perception. A strong negative correlation (r = -0.48) between total performance time and depth perception in the 3D group (median score of five vs. two, p < 0.001) further supports this association. Smith et al. (2014) [16] also reported significantly shorter durations with 3D systems (25 vs. 42 minutes, p = 0.03), citing quicker task completion under stereoscopic vision. These efficiency benefits are strongly corroborated by the most recent literature, including a 2025 prospective study by Subedi et al., which demonstrated significantly reduced procedure durations with 3D systems [17]. Furthermore, a 2024 analysis by Patel et al. found that 3D visualization significantly enhanced operator efficiency and reduced wasted movements, specifically during difficult laparoscopic cholecystectomies [18]. These results suggest a clear efficiency benefit of 3D laparoscopy in elective procedures, a conclusion supported by a meta-analysis by Zhao et al. (2020) [19] (p = 0.002).

However, this benefit is not universal; Wagner et al. (2018) [20] noted reduced benefits among experienced surgeons, suggesting a ceiling effect. Other studies, such as Koppatz et al. (2019) [13], Hanna et al. (1998) [8], and Buia et al. (2017) [21], reported no time advantage, while Tauriainen et al. (2021) [22] found longer 3D times, likely attributed to the inclusion of emergency cases and surgeon inexperience. In the current study, elective case selection and surgeon familiarity with 3D technology likely contributed to the observed efficiency. Notably, outliers with prolonged total performance time (up to 82 minutes) were consistently associated with intraoperative complications, as noted by Yoon et al. (2019) [23], reinforcing that anatomical complexity remains a key determinant of surgery duration regardless of the visualization modality.

Depth perception

Depth perception was significantly superior in the 3D group, with a median score of five compared to two in the 2D group (p < 0.001). This finding corroborates results from Shaikh et al. (2021) [12], Hanna et al. (1998) [8], and Cheng et al. (2016) [24], who all reported enhanced depth perception with 3D systems. Komaei et al. (2017) [4] also noted consistent improvements across multiple studies. The critical role of restored stereopsis has been recently reaffirmed by Subedi et al. (2025), who observed that enhanced spatial awareness translates to more confident anatomical identification [17]. Patel et al. (2024) similarly highlighted that this superior depth perception is particularly crucial when navigating distorted anatomy in complex cases [18]. Our regression analysis confirmed that superior depth perception was directly correlated with shorter total performance time. This advantage stems from stereopsis, where the brain fuses slightly different images from each eye to create a three-dimensional view, thereby enhancing spatial awareness and surgical precision.

Surgeon ergonomics

Eye Strain

While 3D laparoscopy enhances depth perception, it was associated with significantly higher eye strain in this study. This is consistent with findings by Tanagho et al. (2012) [25], Smith et al. (2014) [16], and Hanna et al. (1998) [8], who all reported increased visual discomfort with 3D systems. Shaikh et al. (2021) [12] specifically noted difficulties with refocusing. The strain is likely attributable to the prolonged use of 3D glasses and the cognitive effort required to process stereoscopic images. However, contrasting results exist; Currò et al. (2015) [15] and Gómez-Gómez et al. (2015) [26] found lower discomfort with active 3D systems. These inconsistencies underscore the impact of evolving technology and individual variability in the perception of eye strain.

Wrist and Hand Strain

The 3D laparoscopy system demonstrated notably lower wrist and hand strain compared to the 2D group (median score: two versus three, p = 0.0117). This supports findings by Cheng et al. (2016) [24] and Shaikh et al. (2021) [12], who reported improved precision and reduced instrument-handling effort with 3D laparoscopy. The reduction in physical exertion aligns with recent observations by Patel et al. (2024), who noted that improved 3D visual feedback reduces unnecessary corrective instrument movements, thereby lessening the physical demand on the surgeon's hands [18]. Similarly, Usta et al. (2015) [27] reported significantly reduced strain, although their study focused on novice surgeons. In contrast, Tauriainen et al. (2021) [22] found a non-significant difference. Overall, the data suggest that the enhanced depth perception provided by 3D imaging reduces physical exertion, thereby confirming its ergonomic advantage in elective laparoscopic surgery.

Neck and Back Strain

Despite the observed reduction in wrist and hand strain, no significant difference was found in neck and back strain between the groups (p = 0.8132). This aligns with Buia et al. (2017) [21] and Koppatz et al. (2019) [13], suggesting that spinal ergonomics are influenced more by surgeon posture and table height than by the visualization system. However, other studies by Komaei et al. (2017) [4], Currò et al. (2015) [15], Zundel et al. (2018) [28], and Smith et al. (2015) [16] have reported reduced neck and back strain with 3D systems, attributing this to improved ergonomics and reduced need for awkward positioning. In our study, the uniformly low strain scores across both groups likely reflect the involvement of experienced surgeons adhering to standardized ergonomic practices typical of a tertiary care setting. As highlighted by Koppatz et al. (2019) [13], physical strain tends to be lower among skilled surgeons, reinforcing the importance of technique over technology alone.

Dizziness and Headache

Dizziness and headaches were significantly more prevalent in the 3D group, with a median score of four compared to two in the 2D group (p < 0.001). This supports earlier findings by Hanna et al. (1998) [8] and Buia et al. (2017) [21], who reported greater visual discomfort with 3D laparoscopy. Contributing factors likely include the prolonged use of polarized glasses and the cognitive load of interpreting stereoscopic images. Conversely, Sørensen et al. (2016) [29] found no significant difference in these symptoms, suggesting that factors such as system calibration, surgeon experience, and duration of use may influence the severity of these side effects.

Intraoperative complications

In this study, there was no significant difference in gallbladder rupture rates between the 2D and 3D groups (p = 0.763), a finding consistent with Buia et al. (2017) [21] and Koppatz et al. (2019) [13]. This indicates that while 3D laparoscopy improves spatial awareness, it does not necessarily eliminate the risk of tissue trauma. Recent evidence by Subedi et al. (2025) further supports this, demonstrating comparable safety profiles and complication rates between 2D and 3D modalities in routine cases [17]. Shaikh et al. (2021) [12] and Yoon et al. (2019) [23] similarly found complication rates to be unaffected by the visualization system. Although Tauriainen et al. (2021) [22] reported higher rupture rates in the 3D group, this was likely due to the inclusion of acute cases and surgeon inexperience. Regarding liver bed bleeding, our study found no significant difference (5 ([10%) vs. 7 (14%), p = 0.761), aligning with Tauriainen et al. (2021) [22] and Buia et al. (2017) [21]. These findings suggest that bleeding is more dependent on anatomy and dissection technique than on the visualization modality. In contrast, Bilgen et al. (2013) [10] and Komaei et al. (2017) [4] reported fewer complications with 3D systems, particularly in complex or high-difficulty cases where depth perception is critical. 

Biliary injury and conversion to open surgery

No biliary tract injuries occurred in either group, aligning with the low complication rates reported by Buia et al. (2017) [21]. The absence of such injuries reflects the high surgical expertise and standardized safety protocols at our tertiary care centre. Furthermore, conversion rates to open surgery were identical with one case (2%) in both groups (p = 0.779). This is consistent with Tauriainen et al. (2021) [22] and Buia et al. (2017) [21], who also observed no significant difference in conversion rates. These findings imply that the decision to convert is primarily driven by severe intraoperative pathology, such as dense adhesions or uncontrolled bleeding, rather than the type of visualization system used. However, as noted by Patel et al. (2024), in highly complex anatomical presentations, the improved depth cues of 3D laparoscopy may help mitigate the risk of severe intraoperative errors and potentially lower the threshold for conversion in the hands of less experienced surgeons [18].

Hospital stay duration

Hospital stay duration was identical between groups (mean 2.06 days), consistent with findings by Tauriainen et al. (2021) [22] and Buia et al. (2017) [21]. This suggests that discharge timing is influenced more by institutional protocols and patient recovery than by the operative method.

Limitations and future research

The study’s sample size (n=100) was sufficient to evaluate primary outcomes but may not capture subtle differences in rare complications. This study was conducted at a single tertiary care centre with highly experienced surgeons, reflecting a high-skill environment. Therefore, these efficiency and ergonomic benefits may differ in less specialized settings or among surgeons at earlier stages of their 3D laparoscopic learning curve. Furthermore, we strictly enrolled patients undergoing elective procedures and excluded acute or emergency cases. So, our findings cannot necessarily be extrapolated to acute care settings. Subjective strain assessments used in this study provided valuable insights, though future studies could incorporate objective measures for added precision. However, future studies should incorporate objective biometric measures such as surface electromyography (sEMG) for assessing muscular strain or eye-tracking technology to quantify ocular fatigue for added precision. Further research with diverse surgeon skill levels, varied procedure types, and larger cohorts could enhance understanding of 3D laparoscopy’s broader clinical benefits, aligning with recommendations from Yoon et al. (2019) [23].

## Conclusions

Both 2D and 3D laparoscopy are effective for elective laparoscopic cholecystectomy. 3D systems offer enhanced depth perception and a shorter total performance time, which can benefit high-volume centres and training programs. The reduction in wrist/hand strain underscores the ergonomic advantages of 3D laparoscopy, beneficial for surgeon comfort and precision. However, increased eye strain and dizziness/headaches in the 3D group indicate a trade-off due to stereoscopic visualization and the need to wear polarized glasses, particularly during prolonged procedures. The lack of differences in intraoperative complications and hospital stay shows that in a tertiary care setting where standardized protocols are in use, complication risks are driven by case-specific factors. While 3D laparoscopy is highly valuable for improving efficiency and ergonomics in elective cholecystectomy, the associated visual fatigue highlights a need for continued technological evolution. Advancements such as 4K-3D systems, autostereoscopic 3D displays, and immersive robotic 3D platforms hold significant promise in mitigating these ocular side effects and improving the overall ergonomic profile for surgeons.

## Appendices

Proforma

Case No.

CR No.

Name of the patient:

Age/ Sex:

Father's/Husband's Name:

Occupation:

Address:      

Date of Surgery:

Date of Admission:

Date of Discharge:

**Table 2:** PROFORMA

Case No.	CR No.
Name of the patient:	Age/ Sex:
Father's/Husband's Name:	Occupation:
Address:	Date of Surgery:
Date of Admission:	Date of Discharge:

History of Present Illness: 

· Pain:

· Fever:

· Last episode of Acute Cholecystitis:

· Co-morbidities:

General Physical Examination

· Blood Pressure:

· Pulse:

· Jaundice:

Clinical Findings (P/A Examination)

1. Inspection:

2. Palpation:

3. Auscultation:

**Table 3:** Investigations

Haemoglobin	SGOT
TLC	SGPT
Platelet	ALP
PTI/INR	S. Urea
S.Bilirubin	S. Creatinine
Serum Sodium	Serum Potassium

Ultrasound W/A:

How would you rate the depth perception you experienced during the surgery on a scale of 1 to 5, where 1 is very poor and 5 is excellent?

How would you rate the surgical strain in the surgery you performed on a scale of 1 to 5, where 1 is very low/no strain and 5 is very high? Please answer based on your exertion during the surgery.

Wrist and hand strain:

Neck and back strain:

Eye strain:

Dizziness and/or headache:

Total performance time (From insertion of Veress needle into abdomen to extraction of GB from abdomen):

Intra-operative complications (present or absent)

Gall bladder rupture:

Bleeding from liver bed:              

Biliary tract injury:

Conversion to open surgery:
